# Analysis of Intelligent Transportation Systems Using Model-Driven Simulations

**DOI:** 10.3390/s150614116

**Published:** 2015-06-15

**Authors:** Alberto Fernández-Isabel, Rubén Fuentes-Fernández

**Affiliations:** Departamento de Ingeniería del Software e Inteligencia Artificial, Facultad de Informática, Universidad Complutense de Madrid, 28040 Madrid, Spain; E-Mail: afernandezisabel@estumail.ucm.es

**Keywords:** intelligent transportation system, smart city, sensor, actuator, traffic lights, simulation, model-driven engineering, modeling language, agent-based modeling, code generation

## Abstract

Intelligent Transportation Systems (ITSs) integrate information, sensor, control, and communication technologies to provide transport related services. Their users range from everyday commuters to policy makers and urban planners. Given the complexity of these systems and their environment, their study in real settings is frequently unfeasible. Simulations help to address this problem, but present their own issues: there can be unintended mistakes in the transition from models to code; their platforms frequently bias modeling; and it is difficult to compare works that use different models and tools. In order to overcome these problems, this paper proposes a framework for a model-driven development of these simulations. It is based on a specific modeling language that supports the integrated specification of the multiple facets of an ITS: people, their vehicles, and the external environment; and a network of sensors and actuators conveniently arranged and distributed that operates over them. The framework works with a model editor to generate specifications compliant with that language, and a code generator to produce code from them using platform specifications. There are also guidelines to help researchers in the application of this infrastructure. A case study on advanced management of traffic lights with cameras illustrates its use.

## 1. Introduction 

The concept of *smart city* appears as an answer to the challenges posed by the management of highly interconnected physical and Information Technology (IT) infrastructures in relationship with their communities and environment [1]. The goal is to take advantage of the collective intelligence in such complex systems to get more efficient, sustainable, and liveable environments [2]. Intelligent (or Smart) Transportation Systems (ITSs) [3] play a key role in this context. Their services try to improve traffic regarding security, sustainability, and reduced costs in terms of time, money, energy, and environmental impact. Examples of these services [4,5] are the timely update of traffic data, the request of the equipment needed to assist in emergencies using the data provided by vehicles, or the tracking of freight along its journey. For this purpose, these systems make use of the pervasive presence of networks of sensors and actuators (Sensor Networks, SNs) deployed in people, vehicles, and the environment of *smart places*, and the high availability of data regarding participants in traffic and their activities [6].

The development of these complex systems faces important challenges [3,5,6]. First, their potential effects on the environment and living beings make it difficult to set up suitable controlled environments for experiments. Second, these systems usually comprehend multiple distributed components (both hardware and software), frequently embedded in other elements such as vehicles, phones, traffic lights, or roads. This kind of system has high costs of development, testing, and deployment. Third, there are many interacting elements in a real traffic setting, what makes difficult to validate hypotheses on the behavior of ITSs.

Simulation appears as a useful tool to deal with the previous issues [7]. It allows controlling the relevant variables of the problem, running multiple experiments, and the incremental development of ITSs (*i.e.*, gradually replacing models and software implementations by the actual components and environment). However, this approach also presents some drawbacks [8], mainly concerned with the difficulties to guarantee the alignment between the resulting simulation and the initial abstract model.

Model-Driven Engineering (MDE) [9] has been proposed as a way to overcome these limitations of simulations [10]. In a model-driven development, researchers mainly specify their simulations using models compliant with well-defined Modeling Languages (MLs). These models are combined and refined (adding new information or modifying it) until it is possible to generate code from them. Transformations automate some recurrent modifications of models, code, and other related artifacts. In this way, all the information required to produce the simulation is explicitly represented. This facilitates validating artifacts with theories and crosschecking among experiments. For instance, the same theoretical model of drivers’ behavior can be the basis for simulations in different platforms, different models of traffic flows in a city can be compared with implementations in the same platform, and all of them can be reused and validated in different projects as long as they keep the same theoretical basis or simulation infrastructure.

**Figure 1:**
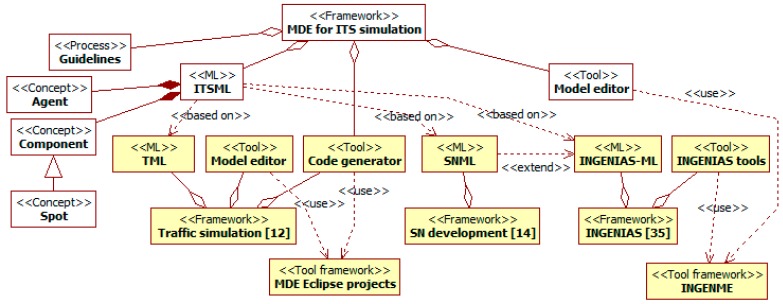
Main components of the Model-Driven Engineering (MDE) framework for Intelligent Transportation System (ITS) simulation (in white) and related works (in yellow). Aggregation relationships (with diamonds) represent whole-part relations, and dependency relationships (discontinuous lines) different relations of use.

This paper presents a MDE framework to develop simulations of ITSs. This framework comprehends: a ML for ITS experts; a *model editor* and a *code generator* tools; and *guidelines* to apply these elements. Figure 1 provides an overview of the framework components and the main related works that influence their development. All considered frameworks adopt MDE approaches, and all their MLs follow the agent paradigm [8,11].

Our ITS ML (ITSML) builds on the foundation of three MLs that address relevant aspects of the problem: the simulation of traffic in [12] (the Traffic ML, TML), and the development of SNs in [13] (the SN ML, SNML), which in turn is partly based on the INGENIAS methodology [14] for agent-oriented software development and its INGENIAS ML (the INGENIAS-ML). The ITSML provides a seamless integration of their concepts (including direct reuse of parts of their MLs) and additional elements. The integration is based on the agent paradigm underlying these languages. Thus, the ITSML distinguishes between *agents* (*i.e.*, intentional and social entities) and *components* (*i.e.*, elements of systems that are not agents).

*Components* have a state, are able to produce events, and make available interfaces with methods to manipulate them. Most concepts from the other MLs become *components* here, e.g., the devices of SNs and the elements of the environment in traffic. The execution of methods can be linked in workflows through the production and use of information (e.g., external notifications or internal facts). This ML also introduces the concept of *spot*, as a *component* that indicates where devices can be placed and what they can perceive and act on. For instance, persons, vehicles, and traffic lights are *spots*.

*Agents* represent both people (e.g., *person* in [12]) and software agents (e.g., *managers* of devices in [13]). They follow a perceive–reflect–act cycle, with specific *components* for each of these tasks. This facilitates decoupling the interaction with the environment and the modification of their internal state.

As general features, the ITSML provides inheritance and instantiation mechanisms. The first one supports the specialization of concept types at the model level. The second one allows defining instances of any type in models, which makes possible the full specification of the simulation at that level. There is also a general association relationship between concepts that can be stereotyped.

The tools of the framework are a model editor developed using the meta-editor INGENME [15], and the code generator from [12]. The first one supports the specification of models compliant with the proposed ML. The second one allows mapping elements in models to code templates that are used to generate the source code of simulations.

The case study that illustrates the use of this infrastructure is the simulation of the management of traffic lights in [16]. That work uses the Simulation of Urban MObility (SUMO) open traffic simulation suite. The paper considers traffic lights that optimize flows in junctions by perceiving the length of their queues and coordinating among them. This case study addresses the development of such simulation using the proposed approach. It shows how most of the simulation can be specified with models and then to generate code from them, which reduces coding effort and facilitates its understanding and later modification. Additional experimentation is also discussed more briefly.

The rest of the paper is organized as follows. Section 2 reviews the background of this work in MDE (see Section 2.1), and the development frameworks for traffic simulations (see Section 2.2) and SNs (see Section 2.3). The framework for the model-driven development of ITS simulations (see Section 3) is described through its ML (see Section 3.1) and its development guidelines and tools (see Section 3.2). Section 4 applies it to the case study of the simulation of intelligent traffic lights (see Section 4.1) and presents additional experimentation (see Section 4.2). The results are compared with related work in Section 5. Finally, Section 6 discusses some conclusions and future work.

## 2. Background

The approach for the development of simulations of ITSs is based on four main works, which are discussed in the following subsections. MDE (see Section 2.1) is the general paradigm for software development used in this research. It is focused on models and their semi-automated transformations. Three development frameworks adopting this paradigm contribute to our research: those for traffic simulations [12] (see Section 2.2) and SNs [13], this extending the INGENIAS methodology [14] (see Section 2.3). The proposed framework integrates elements from these works with specific extensions for ITSs, both at the levels of ML and tools.

### 2.1. MDE

MDE [9] organizes software development around *models*. These gather most of the information required to produce systems and their related artifacts. The development process is mainly conceived as an iterative refinement of models, where information is added to them and modified. *Transformations* automate some of these modifications, and also generate from models artifacts such as documentation, code, or tests. Examples of transformations can be mapping abstract theoretical models to design ones for a given simulation platform, or adding a default goal of path-following to a driver agent. There are alternatives to work with these elements.

In order to enable the automated processing of models by tools, MDE needs to define formally their MLs. Metamodels are the most widely accepted means for this task regarding graphical graph-oriented MLs, which are the most used [9]. A metamodel defines the primitives available in the language, as well as the constraints applicable to them.

Metamodels are specified using meta-modeling languages. The Meta-Object Facility (MOF) [17] is the standard defined by the Object-Management Group (OMG). It is used to define popular standard MLs, such as the Unified-Modeling Language (UML) [18]. However, tool support for MOF is limited, so researchers and engineers frequently resort to better supported alternatives. Ecore [19], from the Eclipse modeling projects, has extensive tool support and is aligned with the subset of MOF known as Essential MOF (EMOF) [20]. MOF-based MLs use *references* to relate concepts. These references are directed binary links with only a few properties (e.g., super-type and containment). This makes them cumbersome sometimes to specify complex relationships with attributes. The Graph-Object-Property-Role-Relationship (GOPRR) [21] meta-modeling language considers a richer set of primitives for this task. Its languages are organized around *graphs* that contain *objects* connected by *relationships*. Relationship ends are *roles*, and they can appear in any number. All the previous elements can have *properties*. Some tools based on GOPRR are Metaedit+ [21] and INGENME [15].

MDE approaches process information automatically using transformations. Most times, their inputs and outputs are models compliant with metamodels, or text-based artifacts (e.g., code or documentation) compliant with grammars [22]. There are multiple ways of implementing these transformations [23], from modules written in mainstream imperative programming languages, to mappings written in specific declarative transformation languages and executed by engines. Examples of the first approach are wizards in [12] and modules in INGENME [15]; JET [24] and ATL [25] are popular Eclipse-supported examples of the second approach.

The organization of the overall development process also has multiple alternatives. One of the best known is the Model-Driven Architecture (MDA) [26] of OMG. It defines three levels of abstraction for its MLs. Computation Independent Models (CIMs) are intended for domain-specific information, here traffic and very abstract views of ITSs. Platform Independent Models (PIMs) describe general computational abstractions, not depending on specific architectures, e.g., sensors, actuators, and controllers of ITSs. Platform Specific Models (PSMs) include technology-related information, e.g., the target simulation platform. INGENIAS proposes two MDE processes, one based on the Unified Process [27] and another on Scrum [28]. They are oriented to different types of project and team. Both use almost the same kinds of diagram over the entire development, independently of the level of abstraction of the stage. In all the considered processes, designers manually modify development artifacts or run automated transformations on them.

Transformations can also be used to translate information among models of MLs compliant with different meta-modeling languages [29]. This is a less frequent type of transformation, and its specification is quite complex.

### 2.2. The Traffic Simulation Framework

The *traffic simulation framework* [12] proposes a MDE approach to develop this kind of simulation. It comprehends the TML to describe theories of traffic, and tools to work with it.

The TML is based on Agent-Based Modeling (ABM) [8]. Its agents are intentional entities that represent *persons*. They are modeled in terms of the *goals* they pursue and the *tasks* they can execute to achieve the former. Internally, these agents follow a perceive–reflect–act cycle [11]. An agent perceives its external environment, uses this information to update its internal state, and then to select a suitable goal and a related task according to it, executes this task, and starts again the cycle. This cycle is modeled using *evaluators* (which perform all these activities but the execution of tasks), and *actuators* (that execute the selected tasks).

Regarding traffic, the TML adopts the Driver–Vehicle–Environment (DVE) [30] model. This considers the dynamic interactions among *drivers* moving in an *environment* using their *vehicles*. In these interactions, the mutual influences among the different components affect their features and behaviors. For instance, the mental workload of a driver largely depends on the weather conditions, which are part of the environment. The TML modifies this basic model to be able to integrate different theories of traffic. Its participants are *persons* (that are also the agents), who can drive *vehicles* or not. *Persons* are characterized in terms of the *knowledge* they have and the features of their *profile*.

The ML uses *methods* to specify the actual effects of the interactions among the previous elements. Methods act as placeholders where indications can be provided on how to update dynamically features. There are default methods for several types of entity. An alternative to model these effects is to use the inheritance mechanisms to provide new types of relationship (e.g., *affect* or *impede*), but this can be difficult to specify and semantics not being clear.

Most of the previous concepts can be decomposed in parts (*i.e.*, *components*). For instance, *Profile* has *PComponent*s, and *Environment EComponents*. They can also be extended through inheritance.

The implementation of this framework is based on Eclipse projects. An Ecore metamodel defines the TML, and related tools are based on the Eclipse Modeling Framework (EMF) [19] and the Graphical Editing Framework (GEF) [31]. The two main tools are a model editor and a code generator.

The model editor allows generating graphically instances of the metamodel, *i.e.*, models. These are drawn as graphs of the concepts and relationships included in the metamodel. This editor is directly generated from the metamodel using the EMF and GEF functionality.

The code generator integrates information from different sources to output the source code of simulations. Its inputs include, among others: the traffic metamodel; models compliant with it; the classes EMF generates from the metamodel; libraries describing the target simulation platforms; code templates for specific parts of the generated code; and mappings from model entities to those templates. A template includes text and placeholders that indicate where and what information from models needs to be inserted. The generator manages all this information with graphical wizards. It is implemented using Java with additional libraries.

### 2.3. The SN Development Framework

The *SN development framework* [13] introduces a MDE approach to develop SNs. It is based on the INGENIAS methodology [14] and its tools for model editing [15], and Eclipse transformations [24,25].

INGENIAS is a MDE methodology to develop Multi-Agent Systems (MASs). MASs are a general paradigm (not only for simulations) where the basic abstraction is the *agent*. As it happens in ABM, INGENIAS agents are intentional and social entities. They execute *tasks* pursuing certain *goals*. These *tasks* can change the internal state of the *agent* or its external environment, or trigger interactions. The environment is modeled using *environment applications*, which produce *events* and have *methods* to act on them. Sometimes, these *events* trigger *tasks*. Interactions exchange information among *agents*.

The INGENIAS-ML supports limited forms of inheritance and instantiation relationships for agents. The latter defines the actual *agent* instances that exist in the system from their types. This is used, for example, to specify the system deployment.

The INGENIAS tools are currently based on the INGENME framework [15] for MDE. INGENME supports the definition of MLs based on its implementation of GOPRR [21]. Its meta-editor allows generating functioning model editors from metamodels. These editors are similar to those of GEF [31], but simpler to describe, as they involve less intermediate models. Additional modules can be integrated in the basic version of these editors. These modules are implemented in Java, and have access to programming interfaces provided by the framework to manipulate both metamodels and their models. Among other tasks, they implement transformations. Text-generation modules are based on templates, as in the traffic simulation framework (see Section 2.2).

The SN development framework extends the INGENIAS-ML to model SNs with the SNML. Its models are organized around *containers*. *Containers* are computational devices where other *resources* run. *Resources* can be pure software *utilities* or *devices* with hardware components (*i.e.*, *sensors* and *actuators*). Communication *channels* linking *containers* are considered a particular type of *resource*. The control of these *resources* is the responsibility of *managers*, which is a role that *actor* agents play. These *managers* can be *passive* or *active controllers*. The first group only listens to notifications from *resources*, while the second group can also invoke their modification methods.

The framework includes basic guidelines on how to model with the SNML and translate these specifications to the INGENIAS-ML. The rest of its development follows an INGENIAS process [27].

## 3. The MDE Framework for ITS Simulation

The framework for the development of ITS simulations comprehends the following elements: a ML (see Section 3.1), and guidelines to apply it with a model editor and a code generator (see Section 3.2).

### 3.1. Modeling Language

**Figure 2:**
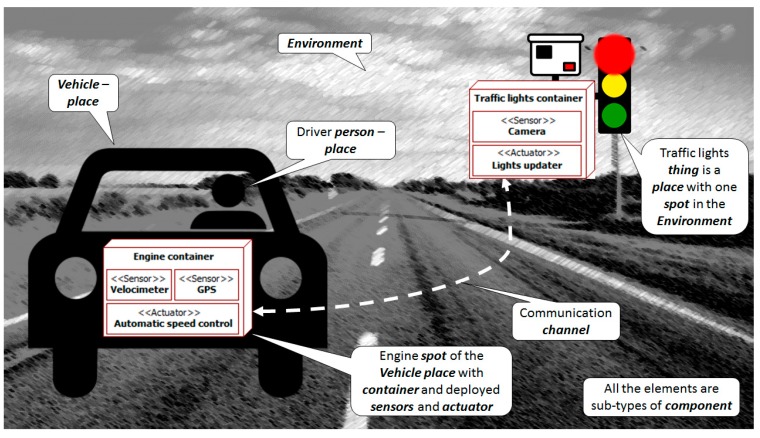
Main concepts (in *italics*) of the ITS Modeling Language (ITSML) in context.

The ITSML is organized around the main concepts that appear in this kind of system and its context (see Figure 2). There are *persons* who interact with *vehicles* and an external *environment*, which is composed of *things*. All these elements are *places* that can contain multiple *spots*. A *spot* is an element that can be observed and/or where *containers* can be located. *Containers* are computational nodes, linked by communication *channels*. In them, software *managers* control the deployed *sensors* and *actuators*. All the previous concepts share some features (e.g., they are modeled with a state and an access interface with methods, and generate events). A common abstract super-concept named *component* gathers these features.

That conceptual basis is the result of integrating and extending multiple researches related to the domain. The ML follows an ABM approach [8], so it makes of agents, and their interactions among them and with external objects, the core of modeling. This perspective is common to several MLs that provide concepts for the ITSML. The TML [12] is the main one, given the close relationship between the domains of traffic and ITS simulations. The INGENIAS-ML [14] offers a rich set of primitives for ABM and basic extension mechanisms of limited inheritance and composition. It is also the foundation of the SNML [13], which models concepts of devices and their controllers in SNs.

The ITSML specifies that conceptual framework using a metamodel. Working with metamodels allows reusing parts of the other MLs. For instance, it supports introducing and modifying concepts and relationships as specializations of those present in the different base metamodels. There are also extensions that mechanisms in the base MLs do not support, but those of metamodels do. For instance, the ITSML has to allow defining instances of arbitrary types in its models.

The meta-modeling language used for the ITSML is the implementation of GOPRR [21] in the INGENME framework [15]. In next diagrams of this section, nodes and relationships, respectively, represent meta-entities and meta-relationships. The ends of these meta-relationships are the meta-roles. Meta-properties appear as attributes or adornments of the previous elements. Examples of these are the numbers in the meta-roles that are cardinality indications. Meta-relationships with triangles represent inheritance, and those with diamonds aggregation, with filled diamonds representing composition and hollow diamonds mere aggregation. This notation is similar to that of UML [18]. As the ITS metamodel integrates concepts from different metamodels, these have been located in packages. Names of concepts and relationships from other metamodels appear qualified with the name of its package, *i.e.*, *traffic* [12], *SN* [13], or *INGENIAS* [14].

The rest of the section discusses in detail the concepts for ITSs in the metamodel. It starts with the general *component* (see Section 3.1.1). Then, it focuses on the *places* where *sensors* and *actuators* can be deployed and with which they interact (see Section 3.1.2). *Managers* control these resources to provide services to *persons* (see Section 3.1.3) moving in an *environment* (see Section 3.1.4). There are also some general mechanisms applicable to all these concepts (see Section 3.1.5).

#### 3.1.1. The *Component* Entity

**Figure 3:**
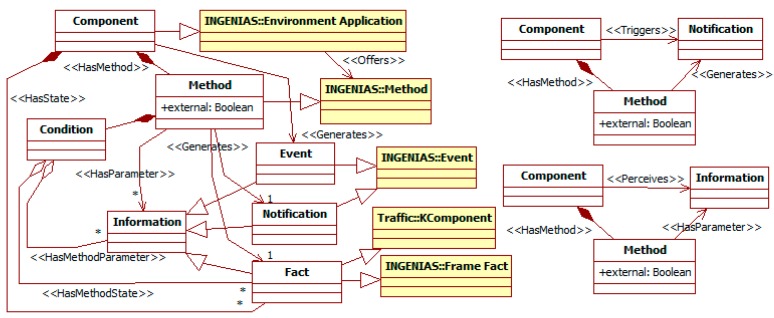
Partial ITS metamodel. *Component* related elements.

Figure 3 is focused on the *component*, which is the base concept in most of the inheritance hierarchies of the ITS metamodel. The SN *device* and the constituents of the traffic *person* (with the exception of the mental entities *goal* and *knowledge*), *vehicle*, and *environment*, become *components*. *Components* have an internal state and an interface composed of *methods*. *Methods* can be *external* (when they can be invoked from outside their *components*), or *internal* otherwise.

The state and methods are characterized in terms of information. Pieces of *information* can be of three types: *facts* make up the internal state; *events* are information that appear in *components* but they do not explicitly produce, and that can be used internally or externally; *notifications* are information produced for external use. *Methods* output both *facts* and *notifications*, but *events* come from non-specified parts of *components*. Besides their results, methods are defined by the parameters they need and the conditions that enable them, which can involve any kind of *information*. *Components* can be connected in pipelines using the *information* their *methods* produce and consume.

The metamodel defines two convenience meta-relationships for *methods* and their related information: *triggers* and *perceives*. The first one relates a *component* to a *notification* that one of its *methods* generates. The second one links a *component* to any *information* that is input to one of its *methods*. These meta-relationships allow focusing on the considered information and omitting the particular *methods* involved.

#### 3.1.2. *Place* and *Device* Related Entities

**Figure 4:**
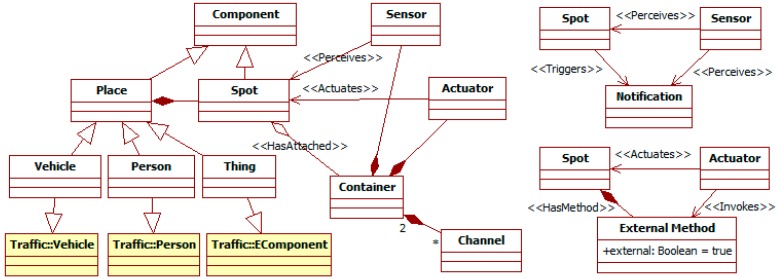
Partial ITS metamodel. *Place* and *Device* related elements.

An ITS needs to gather information from its surroundings and act on them. Figure 4 shows the high-level structure of this context. It is abstracted as a set of *places* that contain multiple *spots*. These *spots* are the *components* that can be actually monitored and modified, and where devices can be placed. Following the DVE model adopted by the TML (see Section 2.2), the participants in the traffic phenomena (*i.e.*, *persons*, their *vehicles*, and the *things* in the external *environment*) are *places*. Their components according to the TML become *spots*.

Following the SNML (see Section 2.3), *devices* are responsible of sensing and acting in the ITS surroundings. The metamodel incorporates the device-related concepts from SNs as *components*. A *container* is the computational resource physically attached to a *spot*. Communication *channels* link them. A *container* runs multiple *devices* (*i.e.*, *sensors*, *actuators*, and *utilities*). Both *sensors* and *actuators* can invoke consult *methods* of *spots*. *Sensors* can also perceive their *notifications*, and *actuators* invoke their external modification *methods*. *Utilities* are pure software components without access to the surroundings.

*Sensors* and *actuators* are thus related to two *spots*, which can be (or not) different: the one to which their *container* is attached (*i.e.*, *HasAttached* meta-relationship); and the one with which they interact. For instance, a *place vehicle* has a *spot* for a *container* with a *thermometer sensor* that measures the *air* temperature, being that *air* a *spot* in the *place weather conditions* of the *environment*. The *perceives* and *actuates* meta-relationships are convenience meta-relationships for these interactions. They link *sensors* and *actuators* with the *spots* they manipulate without considering the involved *methods*.

The control of *devices* can be modeled in their *methods* or using *manager agents*. The activation of a *method* happens when its inputs (e.g., external *notifications* and internal *events* or *facts*) and the internal state of its *device* meet some *conditions*. As a consequence, it generates new information, either internal to the *device* (as *facts*) or external for other components (as *notifications*). For instance, a *timer sensor* in a traffic light has a *method* activated by a *time-consumed event*. When this happens, this *method* produces a *change notification* for the *actuator* in charge of changing lights, which processes it with another *method*. This *method* changes to a target light that depends on a *fact* that represents the current light, and which is part of the internal state of that actuator. On the other hand, *manager agents* (see more on them in Section 3.1.3) control *devices* when complex algorithms are needed (as they have a rich model for decision-making) or there is communication among *containers* (which is one of the specific responsibilities of agents).

#### 3.1.3. The *Person* and *Manager* Entities

**Figure 5:**
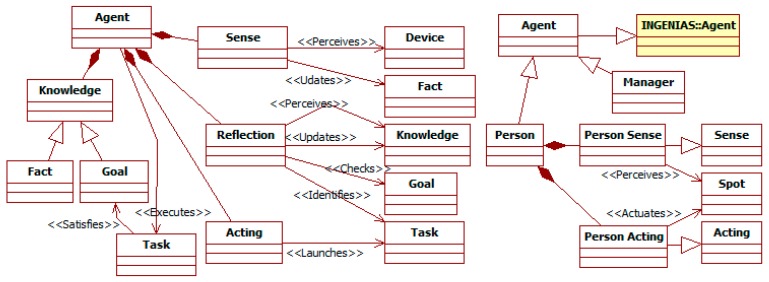
Partial ITS metamodel. *Person* and *Manager* related elements.

The ITS metamodel models with *agents* complex intentional and social components. These include *persons* and *managers* that control *devices* (see Figure 5). The specification of *agents* includes the information they have, their goals and capabilities, and the process to manage the previous elements.

*Knowledge* specifies the internal state. It includes *facts* (*i.e.*, any statement about the world) and *goals* (*i.e.*, states of the world that an *agent* wants to keep or to achieve).

*Tasks* are the high-level actions of agents. They are implemented using *methods* or other *tasks*. When using *methods*, a *task* is eligible for execution depending on the *condition* of the *method*. In this case, the *task* can change the *agent*’s *knowledge*, use *devices*, or act on the environment through *notifications*. Communication among agents is modeled as a particular use of *notifications*. The alternative to define the execution of a *task* is as a workflow of interconnected *tasks* to set up complex activities. These workflows follow the INGENIAS-ML [14].

*Agents* work in a perceive–reflect–act cycle that defines its dynamic behavior. This cycle is modeled with three types of *components* (*i.e.*, *sense*, *reflection*, and *acting*) that operate over their own and their *agent* internal state. The *sense* meta-class models perception as an interaction with observed *devices* that adds or modifies *facts* in the state of the *agent* or the *sense*. The instances of the *reflection* meta-class are responsible of the reflection stage. They analyze available *knowledge* (both *facts* and *goals*), and produce new one or modify the existing. Then, they check non-satisfied *goals*, and identify the *tasks* related to them and suitable for execution in the current context. Finally, the *acting* meta-class represents the actual execution of one of the executable *tasks*.

The *agent* concept is suitable to represent directly control *managers*. For *persons* involved in traffic, there are some modifications. A *person* has specific *person sense* and *person acting components* that allow interacting directly with *spots* without the need of intermediate *devices*.

The previous definitions allow aligning concepts from the ITSML with those of its base languages. The ITSML *agent*, *fact*, and *goal* are sub-types of the INGENIAS-ML meta-classes *agent*, *frame fact*, and *goal*. Both the TML *knowledge* and *profile* are modeled as ITSML *knowledge*, and the TML *goal* is a super-type of the ITSML *goal*. The *person* sub-type of *agent* is also a subtype of the TML *person*.

#### 3.1.4. The *Environment* Entity

**Figure 6:**
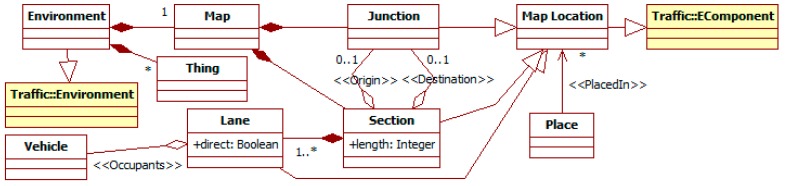
Partial ITS metamodel. *Environment* related elements.

Regarding the *environment*, it represents the elements participating in traffic that are not people, their vehicles, or the ITS components (e.g., *devices* and their *managers*). Examples of its elements are the roads, the traffic signals, the weather conditions, or obstacles. It is an extension of the TML *environment* (see Figure 6). It is modeled as an aggregation of *things*, which are located over a *map*.

The *map* represents the distribution of the physical space. It is a kind of graph composed by a set of *section*, each one linking two *junctions* (at least one) and having a given *length*. This length can be measured in different units, e.g., kilometers or number of vehicle along it. A *section* is a set of *lanes* that can be direct (from the origin to the destination junction) or reverse (the other way). A *lane* has an *occupants* list of references to the vehicles or persons in it ordered according to their position.

The other elements of the environment (the equivalent of the TML *EComponent*) are *things*. They can be placed in specific *map locations*, but also being considered global for the environment, e.g., the weather conditions or the sun position.

*Vehicles* and *persons*, being *places*, can have a location. In the case of instances of *vehicle*, this is mandatory. *Persons* can have a location, when they are pedestrians, or being attached to a *vehicle*.

#### 3.1.5. General Features

The ITS metamodel also includes general instantiation, inheritance, and association mechanisms. For each meta-class of name *name*, there is another meta-class of name *Iname* that represents its instances at the model level. An instance of the later can be linked to an instance of the former with an instance of the meta-relationship *instanceOf*. For example, there is a meta-class *IPerson* linked by a meta-relationship *instanceOf* to the meta-class *Person*, so in a model, a specific driver *IPerson* can be defined as an *instance of* a specific type of *Person*. The inheritance mechanism extends those available in the traffic, SN, and INGENIAS MLs. It can be applied to most of concepts in the ITS metamodel, with the semantics that the sub-concept inheriting from the super-concept has all its features, but it can extend or constrain them with additional elements. Default constraints establish that an animated concept (*i.e.*, an instance of the meta-class *person*) cannot inherit from a non-animated concept (*i.e.*, an instance of a meta-class that is not *person*), and *vice versa*. Regarding association, there is a general meta-relationship *relatedTo* between instances of *component*. It has a *stereotype* property to establish arbitrary types of it.

### 3.2. Development Guidelines and Tools

**Figure 7:**
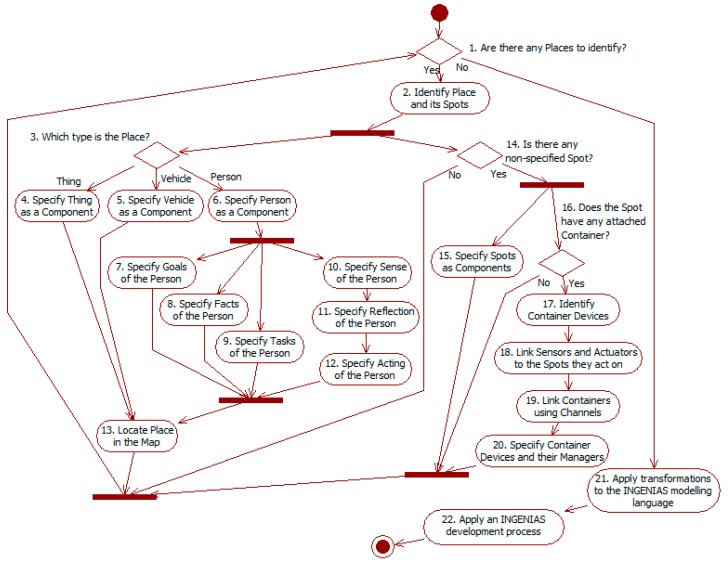
Activity diagram of the development process with the ITSML. Concepts starting with uppercase belong to this ML.

Figure 7 shows the development guideline for the framework. The process has two different stages. The first one (see nodes 1–20) is specific of this work and focused on modeling with the ITSML. The definition of its metamodel (see Section 3.1) provides hints to identify the different concepts. The results of this stage are models that provide an abstract representation of the ITS and its environment. They are independent of specific target simulation platforms. The second stage takes these models as input and runs transformations to get models with the INGENIAS-ML (see activity 21), and then applies an INGENIAS development processes [27,28] to get the simulation code (see activity 22).

The process starts identifying *places* in decision 1. These establish the context of the system and the elements over which the ITS must work. If there is any *place*, activity 2 assigns it a name and identifies its *spots*. Next activities in this path specify the *place* as a *component* (starting with decision 3) and its *spots* (see decision 14). If there are not more *places* to identify, the abstract modeling of the ITS has finished, and the process starts the INGENIAS development (see activities 21 and 22).

The description of a *place* depends on its type (see decision 3): *thing*, *vehicle*, or *person*. All of them have to be specified as *components* (see activities 4–6). This specification indicates the information available from these elements, and the means to interact directly with them. Instances of *person* should not usually include any external *method* to modify its internal knowledge, as it would violate their autonomy according to the agent paradigm. Other of their features can be modified from the environment, for instance to reflect that they have suffered some injury. If needed, these *places* must also be located in the *environment map* (see activity 13), which also implies specifying this.

The specification of a *person* also comprehends its behavioral cycle. This is specified in terms of its *sense* (see activity 10), *reflection* (see activity 11), and *acting* (see activity 12) *components*. *Sense* instances use their *methods* to get *notifications* from external sources (*i.e.*, *spots* and *devices*), and include it as *facts* that are part of the internal state of the *person*. *Reflection* instances model several activities: generation of new derived *knowledge* from that already available; checking *goal* satisfaction according to the current internal state; selection of potential *tasks* to execute according to the state of *goals* and other *knowledge*. *Acting* instances represent the actual execution of *tasks*. Activity 7 is used to specify those *goals*, activity 8 the *facts*, and activity 9 the *tasks*. These elements are linked among them: *tasks* are potentially able to satisfy some *goals*; *goals* are satisfied according to conditions specified in terms of *knowledge*; and *tasks* are eligible depending on the state of associated *goals*.

After specifying a *place*, the process continues with its *spots*. These *spots* are elements where *devices* can be located, or that *devices* can observe and act on. The specification of *spots* starts identifying them (see decision 14). Every *spot* must be defined as a *component* to determine its related *information* and *methods* (see activity 15). *Spots* are also the elements that can have attached *containers* (see decision 16). Activity 17 identifies the *containers* attached to a *spot* and the *devices* running in it. *Containers* are connected through the *channels* identified in activity 19. Among *devices*, *sensors* observe *spots* and *actuators* act on them. These *spots* can be or not the same to which their *containers* are attached. Activity 18 links *sensors* and *actuators* to their target *spot*. Finally, activity 20 specifies the *container devices* as *components* and their *managers* as *agents*, if they exist. Although not included in the figure, the specification of *managers* should follow a similar workflow to that of *person* (see activities 6–12), as both entities are sub-types of *agent*.

Activities 21 and 22 correspond to the transition and the development with an INGENIAS process [27,28]. The first activity develops, if not available, and runs an automated transformation with input ITSML specifications and output INGENIAS-ML models. Then it follows an INGENIAS development process to generate the simulation source code. The main issue here has to deal with code generation. It relies on platform-specific templates (see Section 2.3), which must be available from other projects or need to be developed from scratch.

This approach is in line with MDA [26]. The ITSML models are CIMs and PIMs, and the INGENIAS-ML models are PIMs and PSMs, in both cases depending on their level of abstraction.

Figure 7 shows only a sequential perspective of the process. In fact, its actual application needs to be iterative in its different paths. For instance, in the path from nodes 3 to 13, activities 4–6 can be done in the first iterations to give a high-level description of the system context, while the other activities correspond to a later and more detailed specification. In that path also, when modeling the *person tasks* (see activity 9), it is possible to find out that their execution requires additional internal *methods* of *person*, so activity 6 must be revisited. Moreover, when in activity 18 designers link *sensors* and *actuators* to the *spots* they work on, this can lead to modifications in the definition of those *spots* (made at some moment in activity 15), or even of their *places* (starting in decision 3).

The previous process is supported by two main tools. A graphical model editor allows generating model instances compliant with the ITS metamodel. This editor has been developed using the infrastructure of INGENME [15]. A code generator takes as input the metamodel and the model instances to generate the source code of the simulation for a target platform. Researchers only need to provide the code templates for that platform, and give the correspondences between those templates and the concepts of the ITS metamodel. The code generator has been developed for the traffic simulation framework [12]. It replaces here the modules used in INGENIAS, which offer less assistance in this process.

## 4. Experimentation

This section illustrates the application of the previous framework (see Section 3). It includes a case study (see Section 4.1) and a report on additional experiments (see Section 4.2).

### 4.1. Case Study: A Control System for Traffic Lights

The availability of affordable sensors and actuators for ITSs has made traffic experts reconsider some of the traditional designs of traffic facilities. In particular, traffic signals can be made more *intelligent*, in the sense of being aware of the state of traffic in their areas and coordinate with other signals to improve the overall state in wider areas. The use of *intelligent* traffic lights to control junctions in streets is part of this approach [16]. In that work, the *intelligence* comes from two main features of these signals. First, they are able to perceive the number of cars in the lanes that join in their junctions. They achieve this using Optical Information System (OIS) sensors. Second, they exchange information to adapt their behavior to the overall traffic state in the area with the goal of maximizing the throughput of cars from it. In order to evaluate the benefits of the use of these intelligent traffic lights over traditional ones, the work in [16] simulates both situations using the SUMO platform and real data of traffic. This case study reproduces this experiment using the ITS simulation framework.

The process starts with the identification of potential *places* in the problem (see decision 1 and activity 2). According to its definition (see Section 3.1), a *place* is the component that contains *spots*, which are the elements where *containers* can be attached or that *sensors* can monitor and *actuators* act upon. The presentation of the problem introduces several of these *places*. *Junctions* contain intelligent traffic lights where OIS sensors are located. These sensors perceive lanes from *streets*.

**Figure 8:**
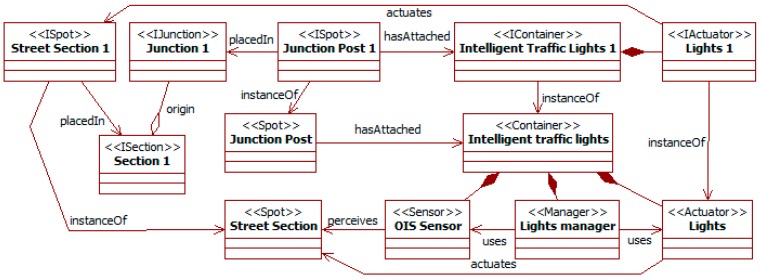
Intelligent traffic lights. *Container* related elements.

At this point, the process can continue by specifying those *places* (starting in decision 3) or the *spots* and related *devices* (starting in decision 14) (see Figure 7). As the original work pays particular attention to the system *devices*, this case study continues in the second path. Figure 8 shows the model instance resulting of it.

Decision 14 begins identifying the *spots* of the previous *places*. The traffic lights are considered as located in the *junction posts* surveying the main *street sections*. Both *junction posts* and *street sections* are *spots*, as the former represent where *devices* are located, and the latter what they survey and affect. Activity 15 specifies these *spots* as *components*. In this case, they do not have any specific state or functionality to specify. Only *street sections* could have it related to the vehicles in them. However, this specification is considered later in the environment *map*.

The next nodes in the path focus on the *devices* the simulated ITS uses to perceive and act. The first step in decision 16 is discovering the *spots* with *containers* for these *devices*. In this case, there is only one of such *spots*: the *junction posts*.

Activity 17 identifies the *devices* running in the *containers*. The *intelligent traffic lights* are modeled as *containers* in *junction posts*. They have as *sensors* the *OIS sensors* that allow them perceiving the queues in their lanes (using the *street sections*); their actuators are their red, green, and yellow *lights*. Activity 18 links *OIS sensors* to the *street sections spots* they watch, and *lights* to the *street sections spots* they are pointing at.

The *containers* of ITSs are not isolated. They set up a complete system thanks to its communications through *channels*. Activity 19 identifies these channels. The *intelligent traffic lights* are connected in this way (not shown in the figure) to share the state of the whole area.

Activity 20 models the *sensors* and *actuators* of *containers* as *components*, and their *managers*. An *OIS sensor* has a method to consult the number of vehicles in its lane. A *light* has a state characterized by its current color, the time remaining in it, and the current default time assigned to each color; its methods allow checking the previous values and changing the periods assigned to each color and which to display. As in the SNML, complex control algorithms are modeled using the *managers* of *devices*, which are agents. In [16], traffic lights adapt the time assigned to each color according to the information from their sensors and from traffic lights in the other junctions. For this problem, a *lights manager* orchestrates work in each *intelligent traffic lights* container. It obtains information from its *OIS sensors* and sends it to the *managers* in the other junctions using the *channels*. It also receives from the *channels* the updates from the other *junction posts*. Finally, with all of this information, it updates the lights state.

Figure 8 also shows how to introduce instances of the types identified for a given problem. The instance *lights 1* of the class *lights* runs in the instance *intelligent traffic lights 1* of the *container*, and it governs the traffic of the instance *street section 1* of the class *street section*.

After modeling *spots*, *containers*, and their *devices*, the specification needs to consider the persons and their vehicles, as well as the environment where all these elements interact. This corresponds in the process to the paths for *places* starting in decision 3 (see Figure 7).

In [16], only vehicles are modeled, as drivers are implicit in their control. Using data from real places, researchers determined the volume of traffic in the different sections of streets and the junction turning percentages. In the ITSML, the *vehicle* is a passive *component*, and the decision-making is modeled with the driver *person*.

Activity 5 models the *vehicle component*. In this case, it has no state. The *environment map* models its position (see later activity 13). Its methods should move the car, change the lane, and check its own position, that of other vehicles around it, and the color of the traffic lights in its *street section*.

The specification of *person* is more complex. It includes the elements representing its basic knowledge and actions (see activities 6–9), and the perceive–reflect–act cycle (see activities 10–12).

Activity 6 specifies the *person* as a *component*, mainly regarding the external methods intended to check its state. Here, no one is needed. Activities 7–9 model its knowledge and capabilities. The *goal* of the driver is the path to follow in the street. It is characterized by the entry and exit points to the street, and the exit to take at each junction. The agent only needs to know the position of the vehicle to make decisions. This is a *fact* that directly translates the information provided by the *vehicle* methods. The *tasks* of the agent are those to check the environment and manoeuver the *vehicle*. In the first group are *checking junction* (to know when to turn and in which direction), *checking position* (to know if there is a *vehicle* in a given position), and *checking lights* (to know if the *vehicle* can go into the junction given the traffic lights). The second group includes *moving forward*, *turn* (left or right and the number of exit), and *stop* the *vehicle*.

Activities 10–12 use the previous elements to represent the acting cycle of the driver *person*. Activity 10 models *sense*. It only needs to *perceive* the *vehicle spot* to assert the *facts* regarding its current position, whether the surrounding positions are busy, and the color of traffic lights. Activity 11 models *reflection*. It compares the current position with the next turn. If the *vehicle* has not reached the turning position and lights allow it, it chooses the *task* to move forward; if it has reached the position, it chooses to turn when the target position is available; in other case, it waits. Activity 12 models *acting*, that translates one of the potential *tasks* for execution to *person* and *vehicle methods*.

The last *places* to describe are *things* in the environment. Activity 4 specifies them as *components*. The already mentioned *junctions* and *streets* are *things*. Here, their specifications are empty because these *places* are only containers for *spots*.

The final step in this modeling is activity 13, where *places* are located in the *environment map*. In [16], there are three junctions across a street, and each junction connects two sections of the street and a transversal section from other streets. The *thing street* is located over the three junctions and two sections. These elements and the transversal sections have direct correspondences in the ITSML as parts of the *map*. The model also needs to specify the number and orientation of *lanes* in each *section*.

**Figure 9:**
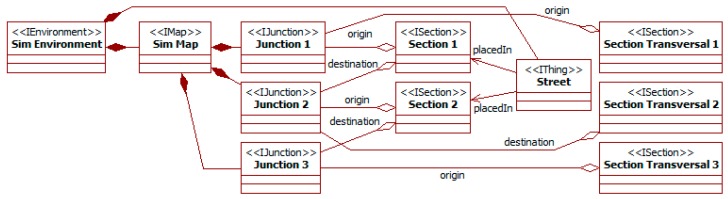
Intelligent traffic lights. *Environment* related elements.

Figure 9 shows the model resulting of these activities, omitting *lanes* of *sections* and the links between the *street* and *junctions*. All the elements appear as instances (*i.e.*, stereotypes *Iname*) of classes (*i.e.*, stereotypes *name*) that are the direct representation of their meta-classes (*i.e.*, *name* of the meta-class). For instance, the metamodel (see Section 3.1) includes a *section* meta-class. *Section 1–2* and *section transversal 1–3* are instances of a class *section* that is a direct instance (without any modification) of the meta-class *section*. The reason of this modeling is that specifying these instances only needs using existing elements, and not adding new state, methods, or relationships.

When all the previous modeling has finished, activity 21 runs the transformations to create INGENIAS-ML models from these ITSML specifications. Their correspondences follow the inheritance relationships of meta-classes in the ITSML to those of the INGENIAS-ML when they exist. For instance, the ITSML *components* in models become INGENIAS-ML *environment applications*, as *component* is a sub-meta-class of *environment application* (see Figure 3). In the case of multiple inheritances, the base target meta-class is that with the closer semantics. For instance, the ITSML *person* inherits from the INGENIAS-ML *agent*, and through *place* and *component* also of the INGENIAS-ML *environment application* (see Figure 3 and Figure 4). However, its meaning is closer to the INGENIAS-ML *agent*. Thus, the transformation generates for a *person* an INGENIAS-ML *agent*, though it also adds additional elements corresponding to it being also a *place*.

Finally, activity 22 follows an INGENIAS process [27,28] to refine those INGENIAS models and generate the source code for the simulation in SUMO [16]. In this case, INGENIAS models mainly add the description of the interactions among *manager* agents regarding the exchange of information from their *intelligent traffic lights*. SUMO specifies most of the components of its simulations using XML files. The main mappings with the original concepts from the ITSML are the following. The *map* of the *environment* is represented in SUMO as a *network*. Its *nodes* and *edges* are, respectively, ITSML *junctions* and *lanes*. The SUMO *vehicle* merges the ITSML *vehicle* and driver *person*. SUMO does not allow controlling the movements of its *vehicles*, as it provides only a limited set of implementations for them. Nevertheless, the path vehicles have to follow can be specified, and SUMO controls that traffic signals are observed. Regarding traffic lights, SUMO supports *actuated traffic lights* for the experiments described in [16]. They can change the time showing each color according to the number of vehicles in the *edges* they control.

### 4.2. Discussion

The proposed framework has been tested in several experiments with the purpose of assessing its components. These experiments considered different contexts in order to evaluate its adaptability to multiple needs regarding simulation. In particular, the ITSML has been used to specify different models for people and types of ITS, and the process and tools also for several target platforms.

Regarding decision-making (see Section 3.1.3), there has been work with the goal-model of INGENIAS [14] and the reactive model for drivers in [32]. Compared to the ITSML, INGENIAS offers additional primitives to model goals with the distinction between hard (with quantitative evaluations) and soft (with qualitative evaluations) goals, and different relationships (e.g., affects, contributes positively, satisfies, and AND and OR compositions). The work in [32] offers an alternative perceive–reflect–act cycle. Its agents consider timed data, and combine for reflection a reactive model and a short-time planner.

The concepts in the previous models are specializations of types already available in the ITSML. For instance, hard and soft goals [14] are sub-types of *goal*, and the short-time planner [32] of *reflection*. Relationships can be represented using instances of the *relatedTo* meta-relationship. Nevertheless, this is a limited approach, as it does not allow introducing constraints. For instance, INGENIAS relations between goals can be modeled, but not constrained with graphical primitives to link only goals.

There is also work with other types of ITS. Smart roads [33] are ITS which SNs are mainly deployed on roads. Their models with the ITSML use specializations of concepts related to devices and managers (see Section 3.1.2) and the environment (see Section 3.1.4). In [33], there are eye cats (a type of *spot*) deployed on road borders (which are *things*). The eye cats hold magnetic trackers (as *sensors*) that perceive vehicles passing near them. *Manager agents* represent the control of these sensors and their communications.

The framework has also been tested with different target simulation platforms. Besides the already mentioned SUMO [16], it has also been tested with MATSim [34] and the platform for traffic simulation in [35]. MATSim mainly defines vehicle movement using paths and additional parameters that influence it, as SUMO does. Thus, the approach described for SUMO in the case study (see Section 4.1) is applicable with minor changes to MATSim. MATSim can also be extended with additional behavioral modules, as the platform in [35]. The latter distinguishes between vehicles and drivers, and supports complex agent-based models for people. Drivers have a basic behavior of path following that can incorporate maneuvers and general constraints on movement as goals. With the ITSML, those constraints are also modeled as *goals*, linked to *tasks* that represent maneuvers. Tasks are related to *conditions* that represent when those maneuvers can be considered for execution. In all the cases, the first tests with these platforms require developing specific code snippets to connect already available code (that for abstract models) with the platform. This also happened in the experiment reported in the case study. These new fragments could later be reused, or adapted with less effort, when experiments continued, and integrated in the code generation tasks.

In these cases, the process points out the ITSML primitives suitable to model the concepts from those models and platforms. However, two aspects remain little described, the choice of relationships and the use of inheritance. Relationships are deduced from the context provided by concepts. The process focuses on the latter, and these determine the allowed relationships. There are no indications on how to choose when several relationships are available beyond their description in the ITSML. There are also sometimes difficulties to design inheritance hierarchies, particularly to establish what concepts must be extended.

## 5. Qualitative Comparative Analysis

This work is related to two main aspects of research in ITSs. It deals with the relevant concepts to model this kind of system, and the approaches followed in their analysis, development, and simulation.

ITSs cover a wide variety of needs [3,5,6,36], e.g., reduction of traffic congestions, toll collection, parking, collision avoidance, or emergency response. However, their architectures and perspectives on the environment present a similar structure. There is a world external to the system that includes, for instance, environmental conditions (e.g., weather, sun position, or daytime) [33,37], roads [33,38], vehicles [37,39], or pedestrians [39]. ITSs include devices (sensors and actuators), which are their “interface” to interact with that environment [6,40]. Using them, ITSs provide their services. These services may require computational resources beyond devices, e.g., servers to store information or allow users to monitor the system and the environment.

The previous facets can be effectively integrated using a view of these elements based on external interfaces able to manipulate their state. In most cases, their inner structure and behavior can be disregarded. For instance, with the exception of works focused on device control (e.g., [39,41]), most of research adopts a high-level view of devices, focused on the data they gather and the services they provide (e.g., [33,38,40]). Such perspective is also applied in the models of environment elements when needed. The only exception is the map of the environment, which is modeled with some kind of graph, e.g., when working with location information from the Global Positioning System (GPS) [38].

The point of view of simulation is slightly different. It needs to integrate concepts to model the objects in the external reality and their behavior, so it can represent their influence in sensors and actuators [42]. For instance, drivers’ behavior is not only an aspect to observe, but also to manipulate in order to test the person and system functioning in multiple circumstances.

Regarding vehicles, simulations need at least to establish partially their path (*i.e.*, origin and destination, and sometimes intermediate points). The specific way in which a vehicle meets this objective offers variations. There are works that consider an strict adherence to the path (*i.e.*, the path constitutes the precise movement instructions) [16], some partially random movements around it (e.g., to decide how to move between two points of the target path) [43], path calculation algorithms (e.g., shortest path under different cost functions [16,34,43]), and specific maneuvers (e.g., car following, lane change, or turns) [43,44] to follow it. Nevertheless, microscopic models (where vehicles are individually modeled) such as [16,34,43,44], could allow modeling more complex and flexible behaviors by replacing the default ones. However, this is not documented in the reviewed works, and it would probably require modifying the code of the considered simulation platforms.

Most of works discard the explicit modeling of drivers, and only consider vehicle control [16,43]. Some references [43,44] include pedestrians, but only as moving objects, similar in their description to vehicles. General traffic studies are more prone to consider these issues. Their models can specify the mental state and features of people, for instance regarding their goals and ability [30], their decision-making [45], or their roles in traffic (*i.e.*, pedestrians, drivers, and passengers) [12].

Both in the case of vehicles and people, researchers lack of support to extend the provided default implementations. For instance, there is little documentation, libraries, base classes for inheritance, or schedulers for multiple tasks, in the platforms to help coding these modifications.

Our ITSML addresses these issues at several levels. It provides an extensible metamodel able to accommodate the different previous modeling approaches. The specification of environment elements and vehicles as objects with state and method interfaces is achieved with *components*. People and vehicle movements are modeled through the specification of *persons*. The case study (see Section 4.1) already includes an example of path following with maneuvers. The adoption of *agents* to represent *persons* goes further and allows describing the wide range of alternative models for people in traffic studies, from reactive to deliberative ones. Moreover, *managers* of devices are also modeled as *agents*, which facilitates representing complex distributed algorithms for ITSs including information management (e.g., goals, facts, probabilities, or uncertainty). Many issues usually considered in code are promoted to models, so researchers can define their requirements using MLs. Then, standard MDE development methodologies translate models to source code. When this translation is done, it provides documentation and reusable artifacts (*i.e.*, models and transformations) for similar implementations. Thus, it provides the basis to build improved support resources for these tasks.

The already discussed conceptual frameworks are intended to be the basis for the analysis of ITSs. They focus on the external interface of these systems (*i.e.*, behavior perceived by users), the environment, and their mutual influences. However, those references do not consider the internal organization of such systems, beyond, for instance, coding constraints imposed by simulation platforms. There are specific works focused on the architecture of ITSs. They usually present abstract models and hints for their implementation (e.g., [33,37]). Other works address low-level aspects of these systems, e.g., control or coordination mechanisms. They are usually focused on specific aspects, e.g., issues of computer vision in [41]. When dealing with such details, the use of simulations is common [7,16,42].

Works such as SUMO [16], DEUS [43], MATSim [34], the Tokyo Virtual Living Lab [44], the test platform in [35], or those reviewed in [42], are traffic simulation platforms. They include libraries to develop and run simulations, and sometimes user interfaces to specify them. Although there are descriptions of their underlying abstract theoretical models, they are black-boxes. A complete understanding of their functioning can require examining their source code when it is available. Literature has already pointed out this issue as one of the main limitations in the use of simulations for research, as it makes difficult guaranteeing the alignment between abstract models and simulations [8].

In most cases, works do not describe the development process followed. There are not explicit guidelines to reproduce modeling, design, or coding. This lack of documentation hampers validation and sharing experiences on development. Moreover, works focus on solving specific issues at a given level of abstraction, e.g., abstract model, architecture, or control of devices. They do not identify ways to integrate that research with complementary works to offer a complete solution to develop ITSs.

The current work adopts MDE to support these tasks. It provides an approach with several abstraction levels intended for different backgrounds. Researchers on ITSs work with the ITSML at the levels of CIM and PIM. Designers of simulations and programmers work mainly with the INGENIAS-ML at the levels of PIM and PSM. In both cases, experts integrate and refine models adding new information and using transformations. For the first group of tasks, this work offers modeling guidelines; for the second, INGENIAS provides a complete development methodology. The overall approach facilitates the explicit representation of all the information involved in the transition from the abstract models to code, which facilitates validation and reutilization.

## 6. Conclusions

This paper has presented a MDE approach for the analysis through simulations of ITSs. The complexity of these systems and the environments where they act implies high development costs. This kind of issues has been addressed in other domains through simulation. However, its application demands means to facilitate the transition from the abstract models of systems to the simulation, and its validation. Usually, researchers make these tasks manually, what is an additional source of mistakes. Adopting MDE approaches, researchers explicitly describe these steps with models and transformations. This facilitates their examination, and therefore validation and testing.

The proposed framework comprehends a ML, guidelines, and tools to develop ITS specifications and simulations. It is intended for the complete development process, focusing effort on the high-level design. Its definition reuses previous research in aspects related to ITSs. The ITSML is built over the TML [12] and the SNML [13], extending them with ITS-specific concepts. All these languages adopt the agent paradigm [11], which facilitates integrating the different perspectives.

The ITSML conceives all the elements in this domain as *components*, which are characterized by their state and a method-based interface. Flows of control and information among components use these methods. ITSs interact with their external environment using a layer of sensors and actuators, which are also *components*. Complex algorithms are modeled with *agents*. These include components for perception, reflection, and acting that manipulate information and goals.

For control activities, *sensors* perceive *events* from the environment, which they transform in internal *facts*. Their *methods* generate *notifications* from those *facts* that are communicated to other *components*, including *manager* agents. These use available information to check *goals* and decide what *tasks* to attempt. The execution of *tasks* invokes *methods*, which can generate new *notifications*. Some of these *notifications* trigger the execution of *actuator methods*, which act on the environment.

The concepts related to traffic are organized following an adapted DVE [30] model according to the structure of the TML [12]. They include common primitives such as *person*, *vehicle*, and *thing* (*i.e.*, environment element). Here, they are linked to the group of concepts corresponding to *devices* and their *manager* controllers extracted from the SNML. This last group models the interface between ITSs and their external environment.

The ITSML also provides mechanisms of inheritance and instantiation of classes in models. The first one allows flexible extensions of the ML without modifying its metamodel. The second one supports the complete definition of the components of the simulation at the model level. In this way, only algorithms need to be specified with code.

The development guidelines, along with the definition of concepts in the metamodel, help designers to build their specifications. They provide a step-by-step process to look for relevant elements in problems, and translate them to modeling primitives. The low-level design and coding follow INGENIAS [14], an existing MDE methodology for MASs. The use of agent concepts in the ITSML and the INGENIAS-ML facilitates the transition between both stages using automated transformations.

The final elements of the framework are the support tools. A model editor based on INGENME [15] supports specifying models compliant with the ITS metamodel. The code generator of the traffic framework [12] is used in the later stages of the INGENIAS process. It replaces the usual INGENIAS tools, because it provides more guidance to work jointly with models and code templates.

This framework has been used in several experiments. The case study developed a simulation of an ITS with intelligent traffic lights for the simulation platform SUMO [16]. The models specified most of the intended simulation. Only algorithms (e.g., using thresholds and timing to change the color of traffic lights, or the methods to interact with SUMO) need to be coded. As most of this code appears in templates that can be reused in code generation for different model elements, the actual coding effort is highly reduced. Changes in the simulation would only affect the model if they add new instances of available classes of entity or relationship. If new classes appear, only the templates for them need to be added before regenerating the simulation. Templates could be reused from other simulation projects. Similar findings appear in the other reported experiments, which considered simulations with extended models of decision-making, other types of ITS, and several simulation platforms.

This framework is still ongoing work with several open issues, some of them already pointed out in experiments in Section 4. First, the metamodel needs to be enriched with additional primitives. Primitives to represent *tasks* ordering or preferences are not available. Also, the specification of *vehicle* parts and their actions is not possible, for instance to represent the angle and acceleration of a vehicle turn in a junction as done in [44]. Second, certain constraints on the model, for example in the number of instances of a class, inheritance relationships, or values of attributes, cannot be expressed. The use of a constraint language such as the Object Constraint Language (OCL) [46] would reduce here the need to resort to code. Third, the development guidelines need to be extended. Now, they indicate a possible ordering of steps and leave for the metamodel definition the indications on how to identify the different modeling concepts. The identification hints should also be transferred to the process specification. Finally, additional experimentation is required to validate the framework. Current experiments are limited in number, the types of ITS modeled, and the considered simulation infrastructures.
